# Subconjunctival silicone oil complicating strabismus surgery

**DOI:** 10.3205/oc000101

**Published:** 2019-04-04

**Authors:** Shailja Tibrewal, Rolli Khurana, Ramesh Venkatesh, Suma Ganesh

**Affiliations:** 1 Paediatric Ophthalmology and Strabismus Services, Dr. Shroff’s Charity Eye Hospital, New Delhi, India; 2 Vitreoretinal Services, Narayana Nethralaya, Bengaluru, Karnataka, India

**Keywords:** strabismus, silicone oil, exotropia, subconjunctival fibrosis

## Abstract

**Purpose:** To report a case of an otherwise simple strabismus surgery that became complex due to the presence of extensive subconjunctival silicone oil.

**Methods:** A 15-year-old boy underwent strabismus surgery for sensory exotropia. He had undergone two prior vitreoretinal surgeries for traumatic endophthalmitis and retinal detachment, respectively. The second procedure involved injection of silicone oil as an endotamponade. During the strabismus surgery, subconjunctival nodules filled with silicone oil were noted. This made the surgery difficult due to episcleral fibrosis. The multiple silicone oil cysts were removed by rupturing or resecting them along with the tenon’s fascia. The extraocular muscles were meticulously isolated and operated using adjustable suture technique.

**Results:** Following surgery, the strabismus was corrected satisfactorily and the patient was relieved of his ocular discomfort and congestion.

**Conclusions:** Subconjunctival leakage of silicone oil can lead to capacious inflammation and complicate strabismus surgery manifold.

## Introduction

Subconjunctival leakage of silicone oil (SiO) is a known complication following vitreoretinal (VR) surgery. While it may be clinically visible on biomicroscopic examination in around 2.7% of cases [1], histopathological evidence has shown it to be present in as high as 33% [2]. In most situations, the leakage is minimal and does not pose many problems. However, extensive leakage can lead to subconjunctival nodules, scarring in episcleral connective tissues, silicone oil underfill, and ultimately discomfort to patients [2]. Few reports have described the occurrence [1], [2], risk factors [3], [4], and management of subconjunctival SiO [5], [6]. We describe a case in which the presence of 360º subconjunctival SiO made a routine strabismus surgery for sensory exotropia difficult. However, the patient achieved excellent ocular alignment and his chronic discomfort was relieved after the removal of the subconjunctival SiO. 

## Case description

**Figure 1:**
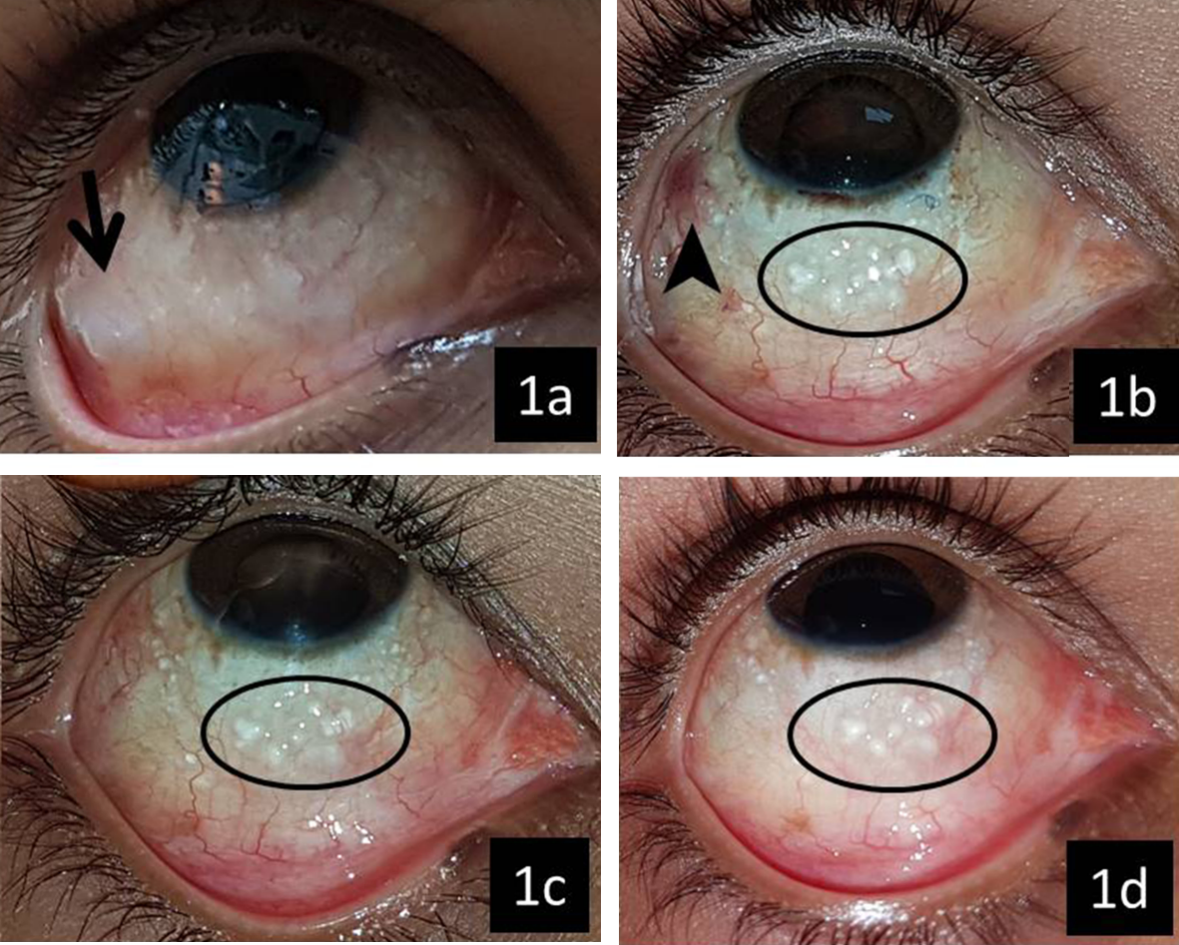
Pre- and postoperative clinical photographs of the conjunctiva of the right eye. 1a: Preoperative photograph showing diffuse congestion and bogginess of the conjunctiva. A large subconjunctival nodule in the inferotemporal area is marked with an arrow. 1b: Postoperative photograph 3 weeks after the strabismus surgery showing marked reduction in conjunctival congestion. The arrowhead shows congestion around the 6-0 Polyglactin suture anchoring the lateral rectus muscle. 1c–1d: Postoperative photographs 3 and 13 months after the surgery. Few persistent small cysts in the inferior quadrant are marked with black circles.

A 15-year-old boy was operated for traumatic endophthalmitis in the right eye in April 2015 with vitrectomy, lensectomy, and intravitreal antibiotics. After three months, he developed inferior retinal detachment for which a repeat vitrectomy, relaxing retinotomy, and SiO (1,000 centistoke viscosity) tamponade were performed. Both procedures were performed through 20 gauge sclerostomies which were sutured with 7-0 polyglactin sutures. The wounds were watertight at the end of the surgeries. He experienced persistent redness and foreign body sensation following the second procedure. He gradually developed outward deviation of the right eye and binocular horizontal diplopia for which he was referred to the strabismus clinic in October 2016. At this visit, his best corrected distance visual acuity in the right eye was 0.8 LogMAR (with +10.25 D contact lens) and near vision was N36 (with +3.0 D add). The conjunctiva was diffusely congested and boggy. Multiple shiny subconjunctival nodules of variable sizes were seen in all four quadrants. They were predominant in the superotemporal and inferotemporal quadrants (Figure 1a (Fig. 1)). Other clinical findings were linear corneal scar superiorly, aphakia, attached retina at the posterior pole, and peripherally detached retina. The intraocular pressure was 10 mm Hg. Orthoptic evaluation revealed exotropia of 25 ΔD with unrestricted ocular movements. A diagnosis of sensory exotropia with sub-conjunctival cysts was made. Lateral rectus (LR) recession and medial rectus (MR) resection were planned which were delayed till May 2017 due to the patient’s unwillingness for further surgery.

**Figure 2:**
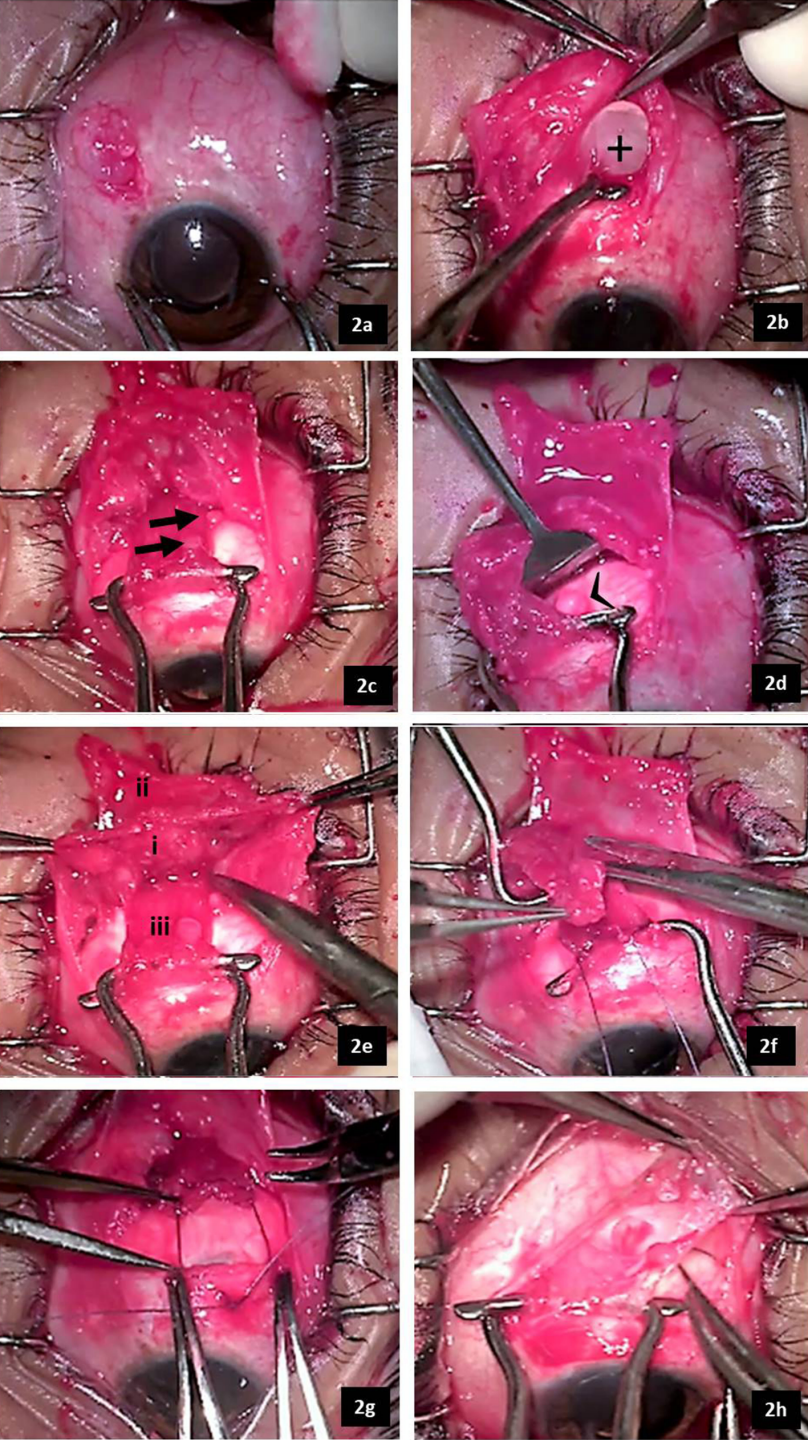
Intraoperative photographs showing the various steps of the strabismus surgery. 2a–g: Intraoperative photographs of the surgical field of lateral rectus recession. 2a: Multiloculated cyst encountered just beneath the conjunctival incision. 2b: Large uniloculated cyst (+) located within the anterior tenon’s capsule at the superior edge of the lateral rectus muscle. 2c: Small SiO cysts (arrows) embedded within the muscle sheath. 2d: SiO cysts located beneath the muscle (arrow head) in the episleral tissue. 2e: Shows the surgical field after separation of tenon’s capsule (i) from overlying conjunctiva (ii) and underlying LR muscle (iii). The thickened tenon’s fascia embedded with multiple small cysts can be appreciated. 2f: Shows resection of tenon’s fascia overlying the LR muscle. 2g: Shows sliding noose adjustable hangback recession of LR. 2h: Intraoperative photograph of the medial rectus resection showing a relatively clear surgical field.

During the surgery, LR muscle was approached under local anesthesia using a paralimbal conjunctival incision. The underlying sclera did not show any thinning or melt. Multiple clear thin-walled oil filled uni- and multi-loculated cysts, ranging from pin-head size to 5 mm, extending up to at least 14 mm posterior to limbus, were encountered below the conjunctiva. They were firmly adherent to the surrounding structures and ruptured on attempted dissection. Numerous cysts were found embedded within the tenon’s capsule, the muscle sheath, and under the LR muscle (Figure 2a–d (Fig. 2)). This had rendered the tenon’s fascia thick and fibrosed (Figure 2e (Fig. 2)). Dissection led to profuse bleeding making visualization and delineation of the muscle onerous and time consuming. The muscle sheath and portion of the tenon’s fascia surrounding the muscle had to be resected to remove the cysts and facilitate isolation of the muscle (Figure 2f (Fig. 2)). The large cysts were removed by rupturing or resecting them. The SiO released from ruptured cysts was thoroughly washed from the ocular surface. After securing the LR with 6-0 polyglactin suture, it was recessed 6.5 mm using a sliding noose adjustable suture technique (Figure 2g (Fig. 2)). Thereafter, medial rectus muscle resection (4.5 mm) was performed (Figure 2h (Fig. 2)). The SiO cysts were sparse in the medial, superior and inferior quadrants. 

In the immediate postoperative period, the patient was orthotropic and did not require adjustment. The conjunctival inflammation gradually reduced (Figure 1b–d (Fig. 1)) eliminating the ocular discomfort. At the last follow-up thirteen months later, his BCVA was 0.8 LogMAR and central retina was attached under the silicone oil. He was diplopia free and was satisfied with the cosmesis.

## Discussion

Extraocular migration of emulsified SiO has been reported to occur in the subconjunctival space [1], [2], [3], [4], [5], [6], suprachoroidal space [7], eyelid [8], orbit [9], and brain [10]. Various factors have been implicated in causing the SiO to enter the subconjunctival space following VR surgery. 

These include

1. multiple surgeries and staphylomatous sclera leading to cheese-wiring of sclerotomy sutures [3],
2. scleral melt following infection or inflammation [9],
3. through glaucoma drainage devices [6],
4. postoperative hypertony causing wound dehiscence [3], [6],
5. unsutured sclerotomies following 23 gauge vitrectomy procedures [5].

In our case, the presence of endophthalmitis and repeated sclerostomies at the same site could have weakened the sclera causing leakage of SiO. However, the sclerotomy sites were sutured after both VR surgeries and there were no spikes of high IOP in the postoperative period. Also, we did not find any areas of scleral thinning or melt during the strabismus surgery. We also speculate that intraoperative factors like overfill of SiO and/or its egress into the subconjunctival space while closure of sclerotomy followed by inadequate irrigation of the spilled oil could have played a probable role. 

In our case, the inflammation incited by SiO in the episcleral tissues led to extensive fibrosis making an otherwise simple strabismus surgery more complex. We were successful in removing the majority of the cysts by resection of the subconjunctival tissues infiltrated by them. We used adjustable suture technique anticipating unpredictable results owing to the scarring around the muscle.

## Conclusions

This case highlights two important points. Firstly, strabismus surgeons should be aware of this complication and anticipate a difficult surgery in eyes with subconjunctival SiO. Secondly, the episcleral tissue fibrosis should be prevented by reducing the spillage of SiO into the subconjunctival space. This can be achieved by meticulous closure of sclerotomy wound, preventing postoperative hypertony and copious irrigation of SiO with saline before closing the conjunctiva. 

## Notes

### Competing interests

The authors declare that they have no competing interests.
